# Increased Metallothionein I/II Expression in Patients with Temporal Lobe Epilepsy

**DOI:** 10.1371/journal.pone.0044709

**Published:** 2012-09-18

**Authors:** José Eduardo Peixoto-Santos, Orfa Yineth Galvis-Alonso, Tonicarlo Rodrigues Velasco, Ludmyla Kandratavicius, João Alberto Assirati, Carlos Gilberto Carlotti, Renata Caldo Scandiuzzi, Luciano Neder Serafini, João Pereira Leite

**Affiliations:** 1 Department of Neuroscience and Behavior, Ribeirão Preto School of Medicine, University of São Paulo, Ribeirão Preto – São Paulo, Brazil; 2 Department of Neurosurgery, Ribeirão Preto School of Medicine, University of São Paulo, Ribeirão Preto – São Paulo, Brazil; 3 Department of Pathology, Ribeirão Preto School of Medicine, University of São Paulo, Ribeirão Preto – São Paulo, Brazil; 4 Department of Molecular Biology, São José do Rio Preto Medical School, São José do Rio Preto – São Paulo, Brazil; University G. D'Annunzio, Italy

## Abstract

In the central nervous system, zinc is released along with glutamate during neurotransmission and, in excess, can promote neuronal death. Experimental studies have shown that metallothioneins I/II (MT-I/II), which chelate free zinc, can affect seizures and reduce neuronal death after *status epilepticus*. Our aim was to evaluate the expression of MT-I/II in the hippocampus of patients with temporal lobe epilepsy (TLE). Hippocampi from patients with pharmacoresistant mesial temporal lobe epilepsy (MTLE) and patients with TLE associated with tumor or dysplasia (TLE-TD) were evaluated for expression of MT-I/II, for the vesicular zinc levels, and for neuronal, astroglial, and microglial populations. Compared to control cases, MTLE group displayed widespread increase in MT-I/II expression, astrogliosis, microgliosis and reduced neuronal population. In TLE-TD, the same changes were observed, except that were mainly confined to *fascia dentata*. Increased vesicular zinc was observed only in the inner molecular layer of MTLE patients, when compared to control cases. Correlation and linear regression analyses indicated an association between increased MT-I/II and increased astrogliosis in TLE. MT-I/II levels did not correlate with any clinical variables, but MTLE patients with secondary generalized seizures (SGS) had less MT-I/II than MTLE patients without SGS. In conclusion, MT-I/II expression was increased in hippocampi from TLE patients and our data suggest that it is associated with astrogliosis and may be associated with different seizure spread patterns.

## Introduction

Zinc (Zn^2+^) is an important modulator of glutamatergic transmission in the central nervous system (CNS) [1], [2], [3]. Zn^2+^ is concentrated in presynaptic vesicles, along with glutamate, and released during normal neurotransmission [4], [5], [6], [7], [8]. Hippocampal neurons are specially rich in vesicular Zn^2+^, particularly in the axonal boutons of granule cells, CA3 and CA1 pyramidal cells and prosubicular neurons [5], [6], [7], [9], [10]. In temporal lobe epilepsy (TLE), one of the most frequent drug-resistant epilepsies in adults, the hippocampus is associated with seizure generation [11], [12]. The intense neuronal activity during seizures can induce high amounts of Zn^2+^ in the synaptic cleft, [13], [14] promoting reactive oxygen species (ROS) production, [15] which can ultimately lead to hippocampal neuronal death [16], [17], [14], [15], [13]. In fact, studies in hippocampi from TLE patients who underwent epilepsy surgery have shown neuronal loss [18], [19], [20], increased glial reaction [21], [22], [23], [24] and reorganization of mossy fibers axon collaterals into the inner molecular layer of the granule cell dendrites [25], [19]. This synaptic reorganization of Zn^2+^-enriched terminals has been hypothesized to contribute to synchronous firing and epileptiform activity [19]. Besides the vesicular Zn^2+^, other intracellular Zn^2+^ pools are present in neurons [26], [27], which can also contribute to neuronal death after an insult [28], [29], [27].

Metallothioneins (MTs) are low molecular weight, cystein-enriched proteins that bound Zn^2+^ and cadmium. They can be found in various tissues, in four isoforms [30]. Isoforms I, II and III are found in the central nervous system (CNS), where the isoforms I and II are expressed in astrocytes and the isoform III is expressed only in neurons [31], [32]. MTs participate in Zn^2+^ homeostasis, scavenging ROS in the brain [33] and stimulate the expression of several neurotrophic and antiinflamatory factors [34]. Studies on rodent models of TLE have shown that MT expression is increased in the hippocampal formation shortly after seizures [35], [36] and that high levels of MTs I and II are associated with reduced neuronal death after seizure-induced damage [37], [36], [38]. However, some studies with neuronal MT (MT-III) indicate that MTs could also contribute to neuronal death in some circumstances [39], [29].

Since MT-I/II levels may be associated with neuron survival after seizures, we hypothesize that MT-I/II expression is altered in TLE and can be associated with the preservation of neuronal density in the hippocampus of TLE patients. Therefore, in this study we evaluated the immunoexpression of MT-I/II and its correlation with hippocampal neuron density in hippocampi of patients with chronic TLE.

## Materials and Methods

### Patients and clinical data

Patients with drug-resistant epilepsy were evaluated at the University of São Paulo Epilepsy Surgical Centre in Ribeirão Preto (Brazil), according to standard protocols published elsewhere [40]. The presurgical evaluation protocol included interviews for epilepsy history, neurological examination, EEG recording, video-EEG assessment, T1- and T2-weighted MRI, ictal and interictal single-photon emission computed tomography (SPECT) scans and neuropsychological tests. Drug resistance was defined according to previous published literature [41].

TLE patients were divided in two groups: (i) mesial TLE (MTLE) and (ii) TLE associated with extrahippocampal tumor or dysplasia (TLE-TD). MTLE group (n = 69) were patients with hippocampal atrophy or with normal hippocampal volume at MRI without other lesions associated with TLE. TLE-TD (n = 17) were TLE patients with tumor or cortical dysplasia in temporal lobe structures other than the hippocampus. From all TLE-TD patients, 4 had non-Taylor focal cortical dysplasia and the remaining had tumors. The tumors observed were grade I ganglioglioma (n = 3), grade I dysembryoplastic neuroepithelial tumor (n = 3), hamartoma (n = 3), teratoma (n = 2), grade III astrocytoma (n = 1) and angioma (n = 1).

For comparison purposes in the neuropathology studies, autopsy controls (Ctrl, n = 19) were obtained from autopsy cases without history of neurological diseases, with no sign of CNS pathologies in *post mortem* pathological evaluation and no history of hypoxic episodes during agony. *Post mortem* time (i.e., time between death and hippocampal fixation) was of 5.15±1.43 hours, ranging from 3.16 to 9 hours. The causes of death were pulmonary insuficiency (n = 6), cardiomyopathy (n = 3), cardiogenic shock (n = 2), sepsis (n = 3), hepatic failure (n = 3), acute lymphoblastic leukemia (n = 1) and gastric adenocarcinoma (n = 1).

Medical records of all evaluated patients were assessed for clinical data analysis. The clinical variables investigated were age at death and cause of death for Ctrl patients and age at surgery, epilepsy duration, age at the first recurrent seizure, seizure frequency per month, presence of secondary generalized seizures, and neuropathological evaluation for TLE patients. This study followed the principles of the Declaration of Helsinki, was registered in Brazilian's Health Ministry and was approved by the Research Ethics Committee of the Hospital das Clínicas, where this study was performed (process HCRP 2634/2008). Written informed consent was obtained from all patients used in this study, and the Research Ethics Committee also approved the Consent Term. Tissue from autopsy cases came from a Brain Bank approved by the Research Ethics Committee of Hospital das Clínicas (process HCRP 9370/2003).

### Tissue collection and histological techniques

Hippocampi from surgery or autopsy were cut in coronal sections and placed in 10% (vol/vol) buffered formaldehyde for one week, followed by paraffin embedding. Immunohistochemistry was performed in 8 µm sections at the level of hippocampal body for evaluation of neuronal, astroglial and activated microglial populations and for MT-I/II expression with antibodies against, respectively, NeuN, GFAP, HLA-DR and MT-I/II. The sections were submitted to endogenous peroxidase blocking with 4.5% H_2_O_2_ in 50 mM phosphate-saline buffer (PSB) pH 7.4, for 15 minutes, followed by microwave antigenic retrieval in 10 mM sodium citrate buffer pH 6.0 (for GFAP) or 50 mM Tris-HCl pH 9.6 (for NeuN, HLA-DR and MT-I/II). After achieving room temperature, the sections went through blocking free aldehyde groups with Tris-glycine 0.1 M pH 7.4 for 45 minutes, followed by blocking buffer with 5% defatted milk and 15% goat serum (#S-1000, Vector) in Triton buffer (PTB, 20 mM phosphate +0.45 M NaCl, pH 7.4, with 0.3% Triton X-100) for four hours. The sections were then incubated with primary antibodies in blocking buffer for 16 hours. We used primary monoclonal antibodies raised in mouse anti-human GFAP (clone 6F2, #M0761, Dako), anti-murine NeuN (clone A60, #MAB377, Chemicon), anti-human HLA-DR (clone TAL.1B5, #M0746, Dako) and anti-equine MT-I/II (clone E9, #M0639, Dako), diluted in blocking buffer at concentrations of 1∶500, 1∶500, 1∶100 and 1∶500, respectively. The primary antibodies were detected using biotinylated rabbit anti-murine IgG (#E0354, Dako), at 1∶200 dilution in blocking buffer, for one hour, followed by revelation with avidin-biotin-peroxidase system (Vectastain Elite ABC kit, #PK6100, Vector) and diaminobenzidine as chromogen (DAB, #34001, Pierce Biotechnology). The development times in DAB solution were 12 minutes for HLA-DR, 10.5 minutes for NeuN and 8 minutes for MT-I/II and GFAP. In order to assure that the different times of fixation of autopsy hippocampi and surgical tissue were comparable, an additional experiment was performed with temporal cortical tissue from one TLE patient. Briefly, a cortical sample was removed during surgery, sectioned in 5 fragments which were kept at room temperature for 1, 2, 4, 6 and 8 hours before immersion-fixation in 10% buffered formaldehyde. Sections of these cortical fragments with different pre-fixation times were mounted on slides and processed in the same manner as the surgical and autopsy hippocampi.

Vesicular Zn^2+^ was evaluated in a subset of cases by neo-Timm histochemistry [19]. Briefly, a fresh hippocampal section was placed in buffered fixative solution (4% glutaraldehyde and 0.1% sodium sulfite) at 4°C for one week, followed by water removal with 20% buffered saccarose for one day. The fragment was dried and frozen in cryostat. Thirty µm sections were utilized for neo-Timm technique, according to previously published protocols [19], [42], [43].

### Immunofluorescence

Colocalization of MT-I/II with neuronal and astroglial markers was performed with the same protocol described above. Endogenous peroxidase blocking and the revelation procedure were omitted. Primary antibodies were raised in mouse anti-equine for MT-I/II (clone E9, #M0639, Dako), in rabbit anti-cow for GFAP (#Z0334, Dako) and anti-human for MAP2 (#sc-20172, Santa Cruz Biotechnology). Sections were submitted to MT-I/II plus GFAP or MT-I/II plus MAP2 incubation, with antibodies diluted in blocking buffer at 1∶100 for MT-I/II, 1∶1000 for GFAP and 1∶50 for MAP2, for 20 hours. The primary antibodies were detected using goat anti-mouse IgG conjugated with Alexa Fluor 488 (#A11001, Molecular Probes) and goat anti-rabbit IgG conjugated with Texas Red (#T2767, Molecular Probes), diluted in blocking buffer, at 1∶300 each, for 2 hours. Following incubation, the sections were submitted to Hoechst 33342 staining (#H1399, Molecular Probes) for 4 minutes, and were mounted in Fluoromount-G (#17984-25, EMS). With this procedure, GFAP and MAP2 were observed in red, MT-I/II in green and cell nucleus in blue. All images were captured in Leica SP5 confocal microscope.

### Histological analysis

Images of all hippocampal regions were obtained with a video monochrome charge-coupled device camera (CCD; Hamamatsu Photonics Model 2400, Japan) attached to an Olympus microscope (Model BX60, Melville, NY), and captured, averaged, and digitized using a frame grabber (Scion Corporation, Frederick, MD) on a Macintosh computer (Model G3, Cupertino, CA). Illumination exposure was uniformly maintained and regularly checked using optical density standards (Kodak, Rochester, NY) in order to prevent any distortion of measurements (immunopositive area, gray level) between the samples. After captured, the image was analyzed using image system software (ImageJ, version 1.37c).

**Figure 1:**
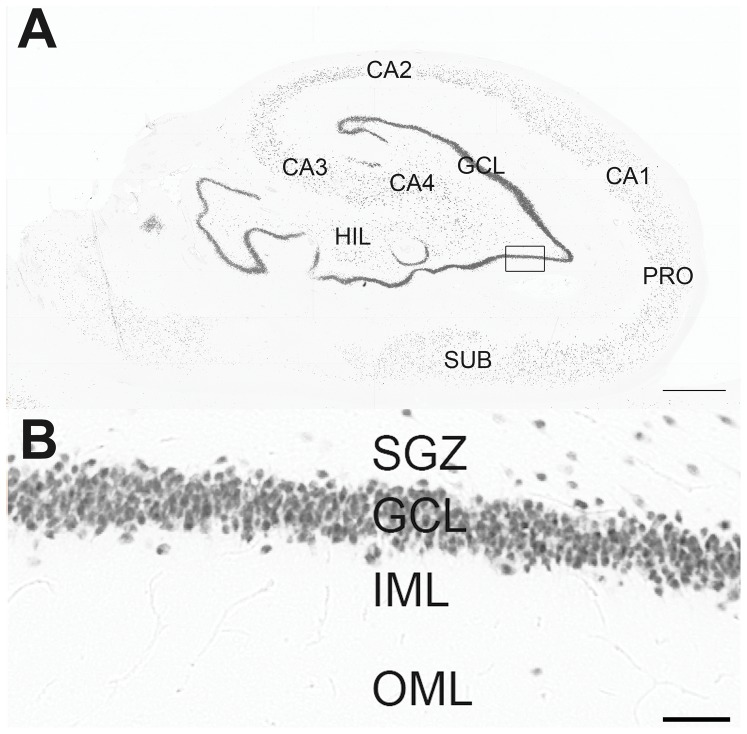
Subfields in the hippocampal formation under NeuN immunohistochemistry. In A can be seen: the granule cell layer of *fascia dentata* (GCL, composed by granular neurons) and the hilus (HIL, composed by several types of interneurons); pyramidal neuronal layers of the hippocampus (CA4-CA1); the subicular formation, composed by prosubiculum (PRO) and subiculum (SUB). In B, a higher magnification of the fascia dentate (marked as a black square in A), composed by subgranule zone (SGZ), granule cell layer (GCL), inner molecular layer (IML) and outer molecular layer (OML). Bar in A indicates 1 millimeter and in B indicates 50 micrometers.

Quantification of the immunohistochemistry was performed with threshold tool, with the investigator blind to the group allocation. After the selection of the region of interest (ROI), the software calculated the immunopositive area by counting all pixels with gray intensity equal or superior to the threshold of staining. A complete protocol for threshold tool can be found at rsbweb.nih.gov/ij/docs/examples/stained-sections/index.html. The threshold was defined for each protein evaluated, based on the mean immunopositivity of all control cases. The evaluated regions were the *fascia dentata* (outer molecular layer, inner molecular layer, granule cell layer, subgranular zone), the hilus and the stratus piramidale of CA4, CA3, CA2, CA1, prosubiculum and subiculum (**Figure 1**). The characterization of hippocampal regions was based on the Lorente de Nó's classification [44]. Results were shown as percentage of immunopositive area/total area.

Additionally, neuronal density was evaluated in the NeuN stained sections. Neuronal count was processed in ImageJ 1.37c software with a 520× magnification for granule cell layer and 260× for pyramidal neurons of CA4, CA3, CA2, CA1, prosubiculum and subiculum. Neuronal densities were estimated with the correction of Abercrombie [45], which permits to estimate the neuronal density through mathematical method, and the results were shown as thousands of cells per cubic millimeter.

Quantification of neo-Timm sections was done by measurement of mean gray value, which varied from 0 to 255, of the hippocampal regions in ImageJ software. The evaluated regions comprised outer molecular layer, inner molecular layer, granule cell layer, subgranule zone and hilus/CA4.

### Statistical analysis

Statistics were carried out in SigmaStat 3.1 software for all tests except for simple regression models, which were performed with SPSS 20. Tests for normality and homogeneity of variances were performed to define data distribution. For parametric variables, One Way ANOVA with Bonferroni *post hoc* or t-test was performed. For the non-parametric variables, Kruskal-Wallis with Dunn *post hoc* or Mann-Whitney tests were used. Fisher's exact test was performed to evaluate categorical data. Correlation between MT expression and cellular populations was performed using the Spearman's test, when n≤30, or Pearson's test, for n>30. Multiple linear regressions were used to define associations between age, neuronal and astroglial populations over MT-I/II expression. All results were considered significant at p<0.05.

## Results

### Clinical data

The clinical characteristics of study participants are summarized in Table 1. The mean age at evaluation was significantly lower in TLE-TD group than Ctrl and MTLE groups (Kruskal-Wallis, p = 0.001). Epilepsy duration was lower in TLE-TD group than in MTLE group (Mann-Whitney, p = 0.002). Recurrent seizures onset (t-test, p = 0.651), minimal seizure frequency in a month (Mann-Whitney, p = 0.397) and frequency of secondary generalized seizures per month (Mann-Whitney, p = 0.557) were similar in MTLE and TLE-TD groups. Fisher's exact test showed that the prevalence of secondary generalized seizures was similar between MTLE and TLE-TD (p = 1.0).

**Table 1:** Clinical history of patients with TLE (MTLE and TLE-TD) and Ctrl cases.

Group	Ctrl	MTLE	TLE-TD	P value
Age at evaluation^1^ (years)	42±16#	38±10#	26±12	0. 001
Epilepsy duration (years)	____	25±10#	15±12	0.002
Age at epilepsy onset (years)	____	13±9	12±7	0.651
Minimal seizure frequency (per month)	____	16±23	25±36	0.397
Number of secondary generalizations (per month)	____	4±7	4±9	0.557
Frequency of secondary generalization (%)	____	59	63	1.000

1 age of death for Ctrl and age at surgery for TLE.

# = statistical difference to TLE-TD; Ctrl = control; MTLE = mesial temporal lobe epilepsy; TLE-TD = temporal lobe epilepsy associated with tumor or dysplasia.

### Changes in immunoreactivity in different fixation times

Quantification of MT-I/II, NeuN, GFAP and HLA-DR immunostaining in sections of cortical fragment in different fixation times revealed that a delay on fixation time was not associated with a decrease of immunoreactivity for all antibodies evaluated (**Figure S1**).

### Neuronal density

**Figure 2:**
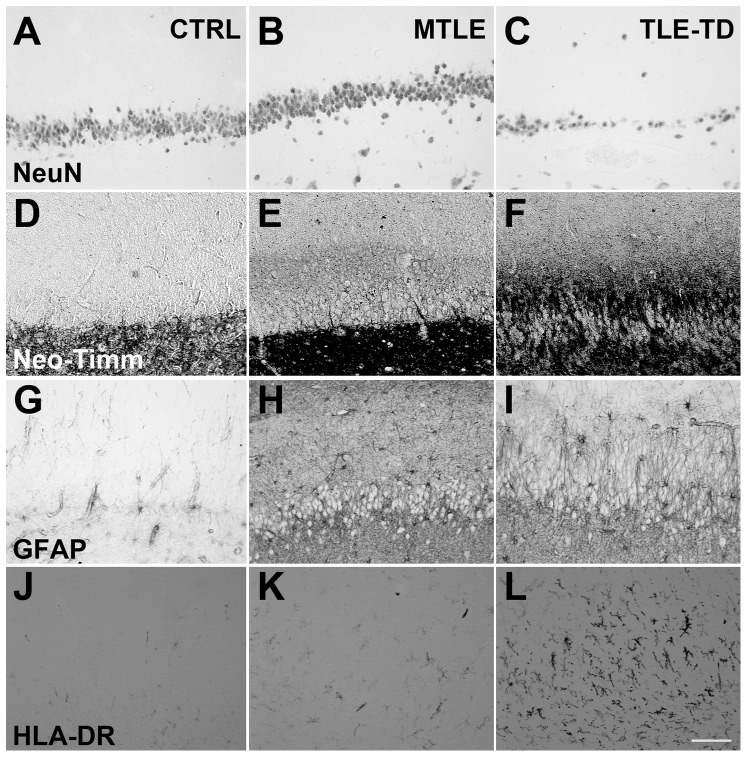
Representative images of NeuN, neo-Timm, GFAP and HLA-DR staining in the *Fascia dentata* of Ctrl, TLE-TD and MTLE patients. The pattern of NeuN staining is the same in Ctrl (A), TLE-TD (B) and MTLE (C) groups, but MTLE shows reduced neuronal population in this subfield. Compared to Ctrl (D), increased neo-Timm staining was observed in the inner molecular layer of *fascia dentata* in MTLE patients (F), but not in TLE-TD (E). As for the astroglial population, both hyperplasia and hypertrophy are observed in MTLE (I) and TLE-TD (H), compared to Ctrl (G). Hyperplasia is also observed in microglial cells in TLE-TD (K) and, more notable, in MTLE (L), compared to Ctrl (J). Bar in L indicates 100 micrometers.

**Figure 3:**
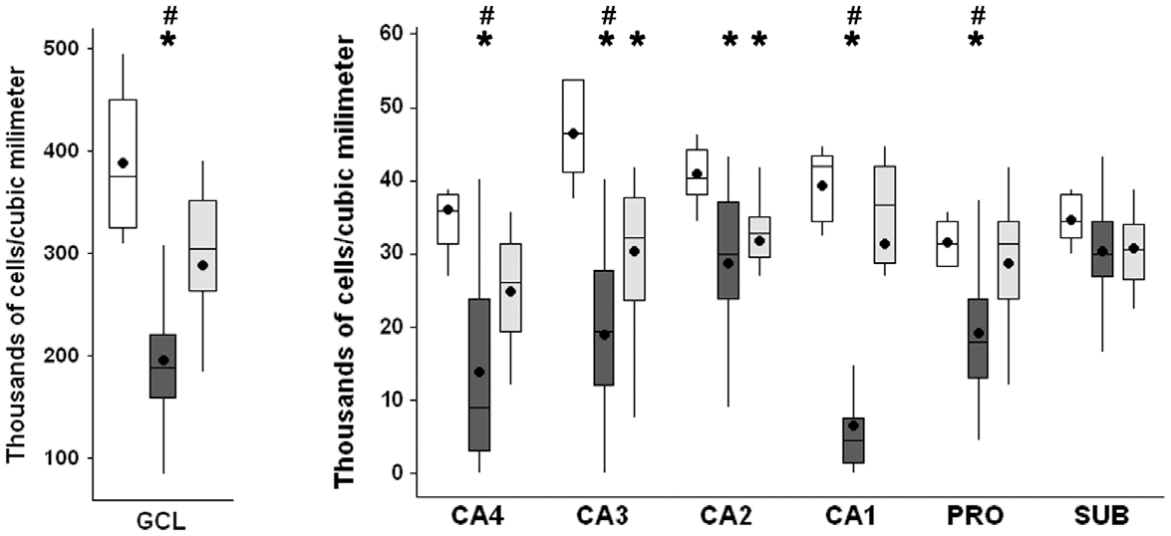
Neuronal density in hippocampal subfields of Ctrl, MTLE and TLE-TD groups. MTLE (dark gray boxplots) had reduced neuronal density (showed as thousands of cells per cubic millimeter), when compared to Ctrl (white boxplots) and TLE-TD (light gray boxplots), in granule cell layer (GCL), CA4, CA3, CA1 and prosubiculum, and in CA2, when compared to Ctrl (p<0.001). TLE-TD presented decreased neuronal density only in CA3 and CA2, compared to Ctrl (p<0.001). The * indicate difference from Ctrl and ^#^ difference from TLE-TD. The dark circles indicate mean.

NeuN immunopositive cells (**Figure 2**) were counted to estimate the neuronal density in the hippocampal subfields. The quantification studies (**Figure 3**) revealed reduced neuronal density in granule cell layer (Kruskal-Wallis, p<0.001), CA4 (Kruskal-Wallis, p<0.001), CA1 (Kruskal-Wallis, p<0.001) and prosubiculum (ANOVA, p<0.001) of the MTLE group, when compared to Ctrl and TLE-TD groups. In CA2 subfield, the neuronal densities of MTLE and TLE-TD groups were reduced when compared to Ctrl (ANOVA, p<0.001). In CA3, MTLE and TLE-TD had reduced neuronal density when compare to each other and to the Ctrl group (ANOVA, p<0.001). No differences in neuronal density were found in the subiculum (ANOVA, p = 0.08). All hippocampal regions of MTLE group showed reduced NeuN immunopositive area when compared with Ctrl, in agreement with neuron density measurements (Data not shown).

### Vesicular Zn^2+^ evaluation

**Figure 4:**
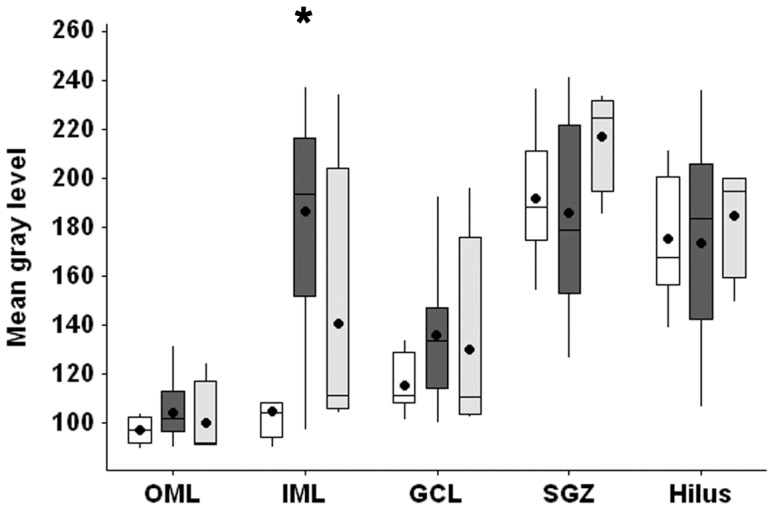
Vesicular zinc staining in the *Fascia dentata* of Ctrl, MTLE and TLE-TD groups. MTLE (dark gray boxplots) had increased neo-Timm staining (showed as gray level intensity), when compared to Ctrl (white boxplots), in the inner molecular layer (IML, p<0.001). No difference was observed between TLE-TD (light gray boxplots) and Ctrl or MTLE. The * indicate difference from Ctrl. The dark circles indicate mean.

Vesicular Zn^2+^ content (**Figures 2** and **4**), estimated by gray value of neo-Timm staining, was increased only in the inner molecular layer of MTLE patients, compared to Ctrl (p<0.001). No differences were observed between Ctrl, MTLE and TLE-TD in the outer molecular layer (p = 0.275), granule cell layer (p = 0.196), subgranule zone (p = 0.467) or hilus/CA4 (p = 0.843).

### Reactive astroglial population

**Figure 5:**
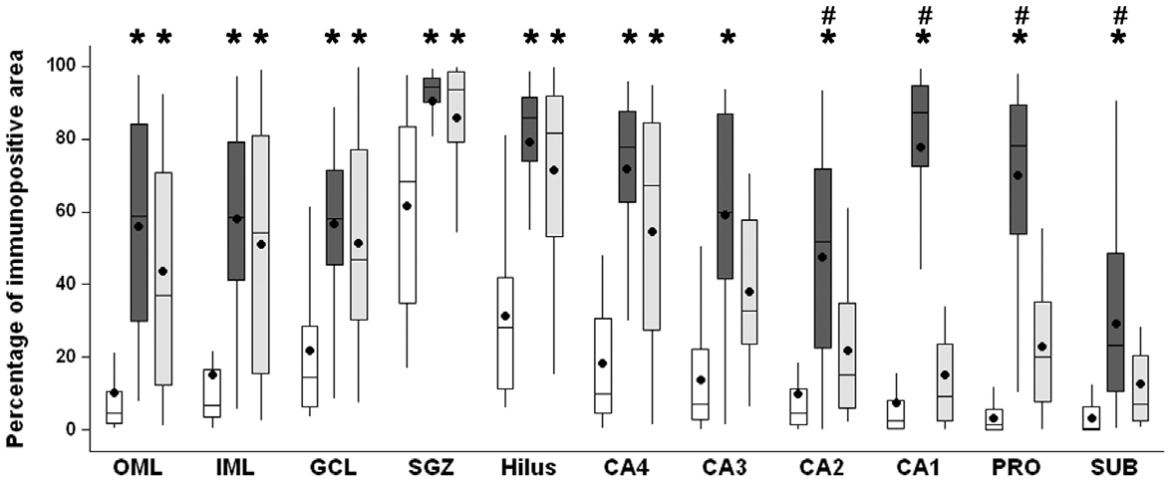
GFAP immunopositive area in hippocampal subfields of Ctrl, MTLE and TLE-TD groups. Compared to Ctrl (white boxplots), MTLE (dark gray boxplots) and TLE-TD (light gray boxplots) groups had increased GFAP immunoreactivity (showed as percentage of immunopositive area) in outer molecular layer (OML), inner molecular layer (IML), granule cell layer (GCL), subgranule zone (SGZ), hilus, CA4 and CA3 (p<0.001), and MTLE groups had increased GFAP immunopositivity in CA2, CA1, prosubiculum (PRO) and subiculum (SUB), compared to Ctrl and TLE-TD (p<0.001). In the subiculum (SUB), TLE-TD had increased GFAP immunoreactivity, compared to Ctrl (p<0.001). The * indicate difference from Ctrl and ^#^ difference from TLE-TD.

GFAP immunopositive area, shown in **Figures 2** and **5**, indicated increased GFAP immnunoreactivity labeling in the outer and inner molecular layers, granule cell layer, subgranule zone, hilus and CA4 of MTLE and TLE-TD, when compared to Ctrl (ANOVA for granule cell layer and Kruskal-Wallis for the remaining regions, p<0.001). In CA2, Sommer sector (CA1 and prosubiculum) and the subiculum, there was increased GFAP immnunoreactivity labeling of the MTLE group, when compared to Ctrl and TLE-TD (Kruskal-Wallis, p<0.001). Increased reactive astrogliosis was also observed in CA3 of MTLE (Kruskal-Wallis, p<0.001), when compared to Ctrl.

### Activated microglial population

**Figure 6:**
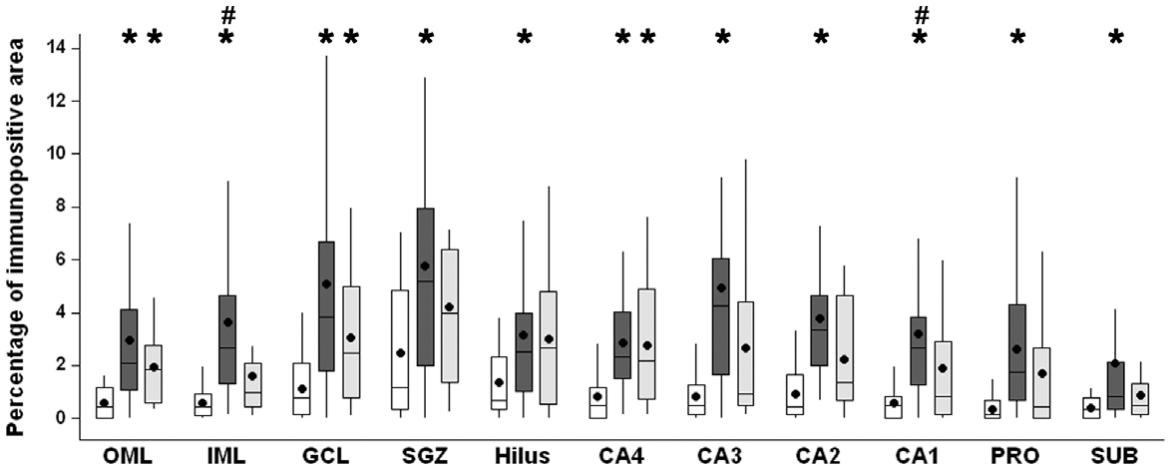
HLA-DR immunopositive area in hippocampal subfields of Ctrl, MTLE and TLE-TD groups. Compared to Ctrl (white boxplots), TLE groups had increased HLA-DR immunoreactivity (showed as percentage of immunopositive area) in outer molecular layer (OML), granule cell layer (GCL), CA4, and CA1 subfields (p<0.001). MTLE (dark gray boxplots) had increased HLA-DR immunoreactivity in inner molecular layer (IML), subgranule zone (SGZ), hilus, CA3, CA2, prosubiculum (PRO) and subiculum (SUB) (p<0.01). In IML, MTLE also presented increased HLA-DR immunoreactivity when compared to TLE-TD (p<0.001). The * indicate difference from Ctrl and ^#^ difference from TLE-TD.

HLA-DR immunopositive area, shown in **Figures 2** and **6**, indicated increased labeling in subgranule zone (Kruskal-Wallis, p = 0.002), hilus (Kruskal-Wallis, p = 0.017), CA3 (Kruskal-Wallis, p<0.001), CA2 (Kruskal-Wallis, p<0.001), prosubiculum (Kruskal-Wallis, p<0.001) and subiculum (Kruskal-Wallis, p = 0.009) of MTLE group, when compared to Ctrl. In outer molecular layer, granule cell layer and CA4 (Kruskal-Wallis, p<0.001) HLA-DR immunopositivity was increased in MTLE and TLE-TD groups, when compared to Ctrl. MTLE group showed increased staining in the inner molecular layer and CA1 when compared to both TLE-TD and Ctrl groups (Kruskal-Wallis, p<0.001).

### Metallothionein I/II immunoreactivity

**Figure 7:**
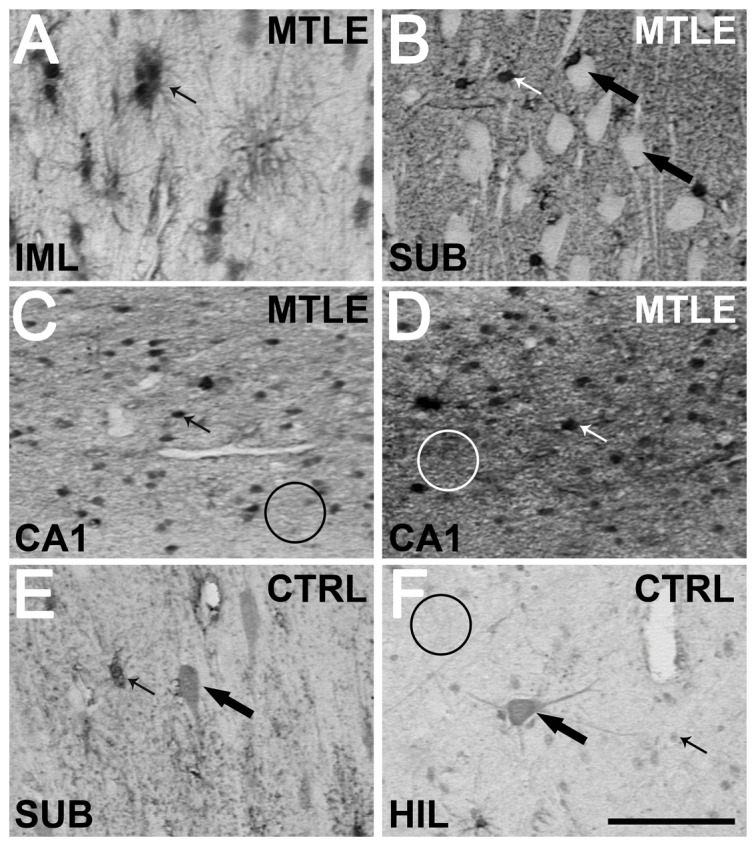
Representative images of MT-I/II staining in several hippocampal subfields. Almost all stained cells have astrocyte morphology (indicated by small arrows in A–F), while neurons remained unstained (white cells pointed by large arrows in B). Only in few cases from Ctrl (E and F) and in one region of one case of TLE were observed cells with neuron morphology (large arrows in E and F). No stained neuron presented the strong staining of astrocytes. In Ctrl, neuropil presented a weak staining (indicated by black circle in F). In TLE the neuropil staining level was heterogeneous, as can be seen in CA1 sections depicted in C and D (indicated by white circles). The representative images shown are from the *fascia dentate* (A), *subiculum* (B and E), CA1 (C and D) and *hilus* (F) of Ctrl (E and F) and TLE cases (A–D). Bar in F indicates 100 micrometers.

**Figure 8:**
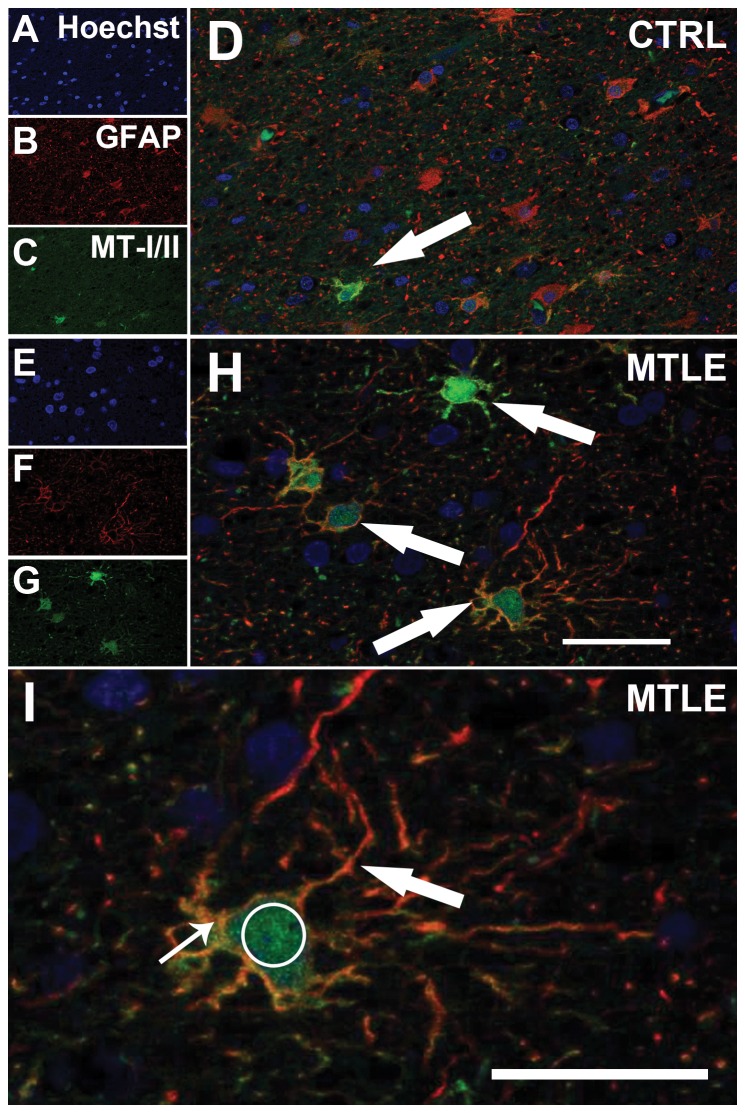
Confocal images of astrocytes expressing MT-I/II in Ctrl and TLE cases. TLE (E–H) patients presented more astrocytes (GFAP immunoreactive cells, red in B, F, D, H and I) expressing MT-I/II (green in C, G, D, H and I, indicated by white arrows in D and H) than Ctrl (A–D). In a detailed view of H (I), MT-I/II expression can be observed in radial branches (large arrow), soma (small arrow) and nucleus (Hoeschst 33342 staining, white circle) of astrocytes. Astrocytes are GFAP immunoreactivity) Bars in H and I indicate 50 micrometers.

**Figure 9:**
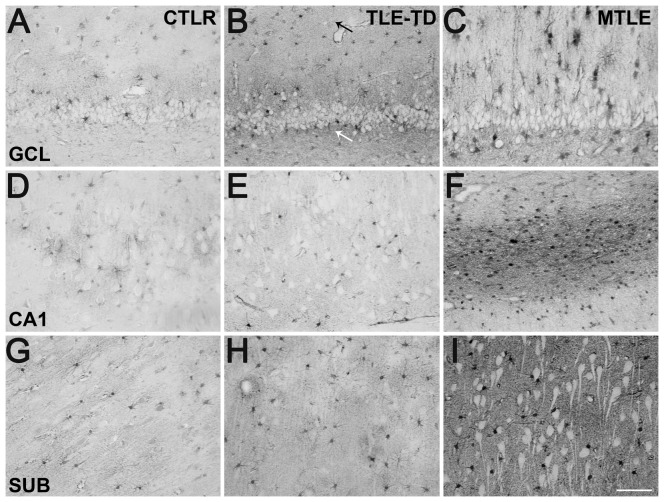
Representative sections of MT-I/II immunohistochemistry in hippocampal subfields from Ctrl, TLE-TD and MTLE patients. MTLE patients had widespread increase in MT-I/II when compared to Ctrl, demonstrated by increased cellular and neuropil staining in C, F and I. In TLE-TD patients, increased MT-I/II expression was observed only in the *fascia dentata* (B) outer molecular layer (small black arrow) and subgranule zone (small white arrow), the entry point of the hippocampus. The representative images shown are from the *fascia dentata* (A–C), CA1 (D–F) and subiculum (G–I). Bar in I indicates 100 micrometers.

MT-I/II staining revealed both cellular and neuropil staining (**Figure 7A–F**). MT-I/II-positive cells had astrocyte morphology, with small round soma and radial processes (**Figure 7A–D**). The staining was present in nucleus, cytoplasm and the proximal portion of the cytoplasmic processes. In two individuals of the Ctrl group and in one MTLE patient, some cells with neuronal morphology and size were also stained for MT-I/II (**Figure 7E, F**). No microglia-like cells were stained for MT-I/II. Neuropil staining showed a granular pattern in all hippocampal subfields (**Figure 7A–F**). Confocal microscopy confirmed the expression of MT-I/II in astrocytes by GFAP-positive labeling (**Figure 8**). A comparison between MT-I/II expression in Ctrl, TLE-TD and MTLE groups is shown in **Figure 9**.

**Figure 10:**
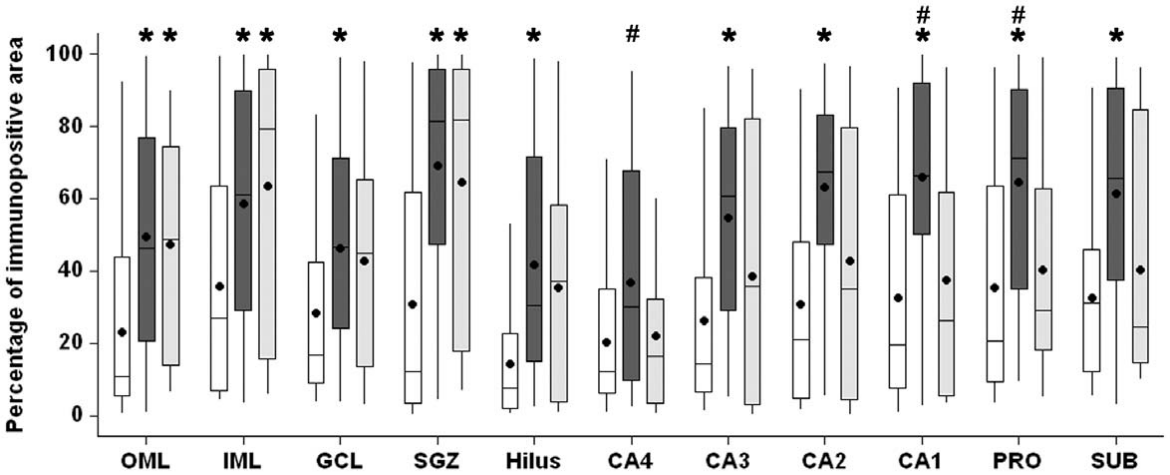
MT-I/II immunopositive area in hippocampal subfields of Ctrl, MTLE and TLE-TD groups. Compared to Ctrl (white boxplots), TLE groups had higher MT-I/II immunopositive area (showed as percentage of immunopositive area) in outer molecular layer (OML), inner molecular layer (IML) and subgranule zone (SGZ) (p<0.01). MTLE (dark gray boxplots) had increased MT-I/II immunoreactivity in granule cell layer (GCL), hilus, CA4, CA3, CA2, CA1, prosubiculum (PRO) and subiculum (SUB) (p<0.05), compared to Ctrl, and also in CA1 when compared to TLE-TD (p<0.001). The * indicate difference from Ctrl and ^#^difference from TLE-TD.

Higher MT-I/II immunoreactivity area (**Figure 10**) was observed in both TLE groups, when compared to Ctrl group. The increase in MT-I/II immunoreactivity area observed in TLE was due to an increased number of MT-I/II-positive cells and to increased neuropil staining. MTLE group showed increased immunopositive area when compared to Ctrl in granule cell layer (Kruskal-Wallis, p = 0.028), hilus (Kruskal-Wallis, p<0.001), CA3 (ANOVA, p = 0.003), CA2 (Kruskal-Wallis, p<0.001) and subiculum (Kruskal-Wallis, p<0.001) and in CA4 when compared to TLE-TD (Kruskal-Wallis, p = 0.041). Both MTLE and TLE-TD groups had increased MT-I/II immunopositive area when compared to Ctrl in outer molecular layer (Kruskal-Wallis, p = 0.002), inner molecular layer (Kruskal-Wallis, p = 0.023), and subgranule zone (Kruskal-Wallis, p<0.001). In CA1 and the prossubiculum, the immunopositive area was increased in MTLE when compared with both TLE-TD and Ctrl (ANOVA, p<0.001).

### MT-I/II immunoreactivity and seizures

**Figure 11:**
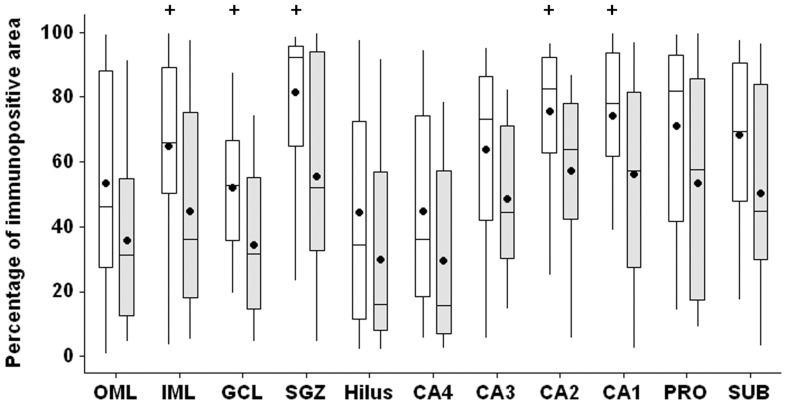
MT-I/II immunopositive area in MTLE patients without and with secondary generalized seizures. Patients without secondary generalization (white boxplots) present increased MT-I/II immunopositivity (p<0.05) in the inner molecular layer (IML), granule cell layer (GCL), subgranule zone (SGZ), CA2 and CA1, when compared with patients that present secondary generalization (light gray boxplots). The ^+^ indicates difference between the groups.

In MTLE group, patients without secondary generalized seizures (SGS) had increased MT-I/II immunopositivity, when compared with patients with SGS, in the inner molecular layer (t-test, p = 0.037), granule cell layer (t-test, p = 0.018), subgranule zone (t-test, p = 0.004), CA2 (Mann-Whitney, p = 0.039) and CA1 (t-test, p = 0.043) (**Figure 11**). No differences in neuronal, astroglial or microglial populations were observed between MTLE patients with or without SGS. In TLE-TD patients, no differences in hippocampal MT-I/II immunopositivity, neuronal, astroglial or microglial populations were observed between patients with and without SGS. Frequency of seizures did not correlate with MT-I/II immunopositivity in all hippocampal subfields.

### Correlations between MT-I/II immunoreactivity, cellular populations and vesicular Zn^2+^

Considering all TLE patients, correlation analysis revealed that MT-I/II immunoreactivity correlated with GFAP immunoreactivity in CA4 (r = 0.312; p = 0.012; n = 65), CA2 (r = 0.275; p = 0.038; n = 57) and CA1 (r = 0.319; p = 0.004; n = 78) and with NeuN in CA1 (r = −0.241; p = 0.034; n = 78). No correlation was found between MT-I/II immunoreactivity and HLA-DR immunoreactivity or neo-Timm staining. In CA4, multiple linear regression revealed a trend to association between MT-I/II expression and GFAP immunopositivity (r = 0.347; p = 0.061, with p = 0.753 for NeuN, p = 0.02 for GFAP and p = 0.111 for age; n = 53). In CA2, multiple regression model revealed that MT expression was significantly explained by GFAP and age (r = 0.574; p<0.001, with p = 0.533 for NeuN, p = 0.018 for GFAP, p<0.001 for age; n = 55). In CA1, MT-I/II expression has a trend to be explained by increased GFAP immunoreactivity (r = 0.364 ; p = 0.015, with p = 0.817 for NeuN, p = 0.069 for GFAP and p = 0.107 for age; n = 77). In summary, in some hippocampal subfields (CA4, CA2, and CA1) there was a positive correlation between MT-I/II immunoreactivity and GFAP immunoreactivity. Different regressions models did not provided a best fit for any of the variables evaluated.

In TLE-TD, there was a positive correlation between NeuN and MT-I/II expression in CA4 (r = 0.543; p = 0.0353; n = 15). No correlation was observed between MT-I/II expression and GFAP, HLA-DR area or neo-Timm density in TLE-TD. Multiple linear regression model was not significant in CA4 (r = 0.590; p = 0.179; n = 15), but NeuN was significantly associated with MT-I/II in this region (p = 0.046 for NeuN, p = 0.662 for GFAP and p = 0.486 for age). For the relation between neuronal population and MT-I/II expression in CA4, the quadratic model provided a better fit, when compared to the linear model (r^2^ = 0.48 and p = 0.014 for the quadratic model versus r^2^ = 0.333 and p = 0.019 for the linear model)

In MTLE, MT-I/II immunoreactivity area correlated with GFAP area in CA4 (Pearson's test; r = 0.319; p = 0.0241; n = 50). No correlations were observed between MT expression and NeuN, HLA-DR or neo-Timm in MTLE. Multiple linear regression revealed no significance in CA4, although GFAP expression was significantly associated with MT expression (r = 0.332; p = 0. 175, with p = 0.703 for NeuN, p = 0.042 for GFAP and p = 0.269 for age; n = 46). No other regression model than the linear provided a best fit for the variables evaluated.

## Discussion

In the present study, we found an increased MT-I/II expression in all hippocampal subfields of MTLE patients and in the *fascia dentata* of patients with TLE-TD. In MTLE patients, MT-I/II expression correlated with astroglial population but not with neuronal population. In TLE-TD group, MT-I/II expression correlated positively with neuronal population only in CA4. In the CNS, MT-I/II are expressed mainly by astrocytes [46] and, when the tissue suffers an injury, increased MT-I/II expression is observed in astrocytes and microglias [46], [32]. In our study, an increased expression of MT-I/II was observed in astrocytes and occasionally in neurons of autopsy and TLE patients. Confocal microscopy in our TLE patients corroborated the finding that MT-I/II are expressed by astrocytes. We also observed an increased expression of MT-I/II in the neuropil of TLE patients. Studies in tissue obtained from animal models of CNS injury have shown that increased MT-I/II expression in the neuropil is most likely the result of higher release of MT-I/II from the astrocytes [47], [48]. Therefore, our data support the notion that MT-I/II changes are essentially related to astroglial population.

Gliosis is a common finding in TLE [21], [22], [23], [24] and is associated with the degree of neuronal death [22], [49], [23], [24]. Similarly with MT-I/II expression, gliosis was more intense and widespread in MTLE than in TLE-TD groups. Furthermore, correlations between the degree of astrogliosis and the expression of MT-I/II observed in TLE patients indicate that MT-I/II expression in TLE is a phenomenon associated with the astrogliosis and, consequently, with the degree of tissue damage. In agreement with this hypothesis, an association between the severity of tissue damage and the increase in MT-I/II expression has been reported in mice subjected to soman-induced SE [35].

Studies in rodents with kainic acid-induced SE showed an association between MT-I/II expression and neuronal survival. Transgenic mice over-expressing MT-I/II have reduced neuronal death, compared to wild type animals [38]. In addition, mice with reduced MT-I/II expression [36] or in knockouts for MT-I/II [37] had increased neuronal death following SE, compared to wild type mice. In our study, MT-I/II expression correlated positively with neuronal population only in CA4 of TLE-TD patients. In MTLE group, where neuronal death and MT-I/II expression are more pronounced, no correlation between neuronal death and MT-I/II was observed. These findings contradict the hypothesis that an increased MT-I/II expression could be related with neuronal survival. Different mechanisms contribute to neuronal death that occurs in the hippocampus of MTLE and TLE-TD patients. In TLE-TD patients, evidence has been shown that neuronal death is a consequence of the recurrent seizures [50]. Although neuronal death in MTLE can also be caused by recurrent seizures [50], the bulk of neuronal death is rather a consequence of an initial precipitating insult (IPI), which usually occurs several years before the epilepsy onset [50], [51]. The neuronal death is also severe in MTLE, often resulting in hippocampal sclerosis, while TLE-TD patients generally have preserved neuronal density [18], [52]. In addition, data indicate that hippocampal atrophy may be determined by a strong genetic predisposition and occur in individuals who never had seizures [53]. Therefore, it is possible that the differential increase in MT-I/II expression in TLE-TD and MTLE is also the result of the different mechanisms associated with neuronal death in such epileptic syndromes.

According to other studies, MTs could also be responsible to neuronal damage and death following SE. In mice knockout for ZnT3, a protein responsible to stock Zn^2+^ in synaptic vesicles, SE increases damage in CA1 [39], [28], [29] and other cerebral regions [29], when compared to wild type mice. In these knockout mice lacking vesicular Zn^2+^, damage in CA1 can be prevented by chelating extracellular Zn^2+^ [28], [29] or by knocking out MT-III gene [39], [29]. However, knocking out MT-III gene in mice with [54] or without vesicular Zn^2+^ [29] increases damage in CA3 after SE. Since all studies that associated MT-I/II with neuronal survival after SE studied mainly the CA3 region, where MT-III is also known to protect from damage [54], [29], one could argue that, in CA1 and other hippocampal regions, MT-I/II could cause damage, similarly to MT-III. We did not find any positive association between increased MT-I/II expression and reduced neuronal population in all hippocampal subfields. Furthermore, mice with reduced levels of MT-I/II [36] have increased damage in CA1 after SE. It is known that MT-I/II binds Zn^2+^ more strongly that MT-III [55], [56]. These observations make us believe that MT-I/II do not contribute to the neuronal damage observed in the hippocampus of TLE patients. Further studies must be performed to better address this issue.

Several developmental studies have indicated that MT-I/II levels increase with the age, [57], [58], [59], [60], [61], [62], [63], [64]. On the other hand, reduced MT-I/II expression has also been reported in the adult rat brain when compared to young brain [65], and no differences were observed in aged and adult brain specimens of rat [66] and calf [67]. Also, it is already known that longer epilepsy duration can increase the neuronal death observed in hippocampal sclerosis and is associated with the neuronal death in non-sclerosis cases [50]. Therefore, we must also account for age and epilepsy duration as factors for the changes observed in MT-I/II expression. We did not see relation between epilepsy duration and MT expression in our multivariate analysis. However, in some regions, age at evaluation was significantly associated with MT-I/II expression. For example, in CA2 of all TLE patients and in CA4 of TLE-TD age at evaluation predicted MT-I/II expression. Although our findings indicate that age can contribute to the increased MT-I/II expression observed in TLE, the pathological changes associated to the epileptic condition (i.e., gliosis and neuronal death) are still the main factors related to the increased MT-I/II expression in the hippocampus of TLE patients.

Reorganization of vesicular Zn^2+^ in the hippocampus is often observed in TLE [19], [20], and Zn^2+^ can trigger MT-I/II expression [59]. Then, it is also important to consider the effect of the Zn^2+^ pool over MT-I/II expression. In agreement with other studies [68], [69], [19], we only observed significant increase in vesicular Zn^2+^ in the inner molecular layer of MTLE patients. No correlation was observed between MT-I/II expression and vesicular Zn^2+^ in our TLE cases. This does not exclude an association between MT-I/II expression and Zn^2+^, provided that only 10% of all Zn^2+^ in the brain is located in vesicles [70], [7] and only a small fraction of the Zn^2+^ released during neurotransmission will reach the astrocytes to induce MT-I/II expression.

Data have shown that the increased MT-I/II immunoreactivity observed in animal models of TLE can also be a factor associated with the seizure generation process. Transgenic mice over-expressing MT-I, have increased seizure duration, a tendency to reduced latency, but similar number of seizures after kainic acid administration [38]. Since MT-I/II act chelating free Zn^2+^ [31], [14] and Zn^2+^ chelation increases tissue excitability and facilitates seizure generation [71], excessive MT-I/II levels can reduce free Zn^2+^ in the synaptic cleft, increasing neuronal excitability and affecting seizure generation. Our data showed a similar frequency of seizure between MTLE and TLE-TD patients. In agreement with previous studies, we found no correlation between seizure frequency and MT-I/II expression in TLE [38].

In MTLE, we found increased levels of MT-I/II expression in patients without SGS, when compared with those with SGS. This could indicate that MT-I/II expression is associated with different seizure spread patterns from the epileptogenic hippocampus to other brain regions. It is important to point out that no difference in neurons or glial cells was observed between MTLE with and without SGS. Studies from different groups also observed no association between changes in the hippocampus and SGS [72], [73], [74]. These observations suggest that the increased MT-I/II expression in patients without SGS is not an effect of gliosis, but it is independently associated with SGS. Further studies with animal models of TLE should evaluate more closely the relationship between MT-I/II expression and seizure susceptibility.

The differential pattern of increase in MT-I/II expression in MTLE and TLE-TD patients may also be associated with the site of seizure generation. Seizures are known to induce MT-I/II expression in the epileptic hippocampus [75]. In MTLE patients, where MT-I/II increase was widespread, most focal seizures are generated within the hippocampus [76]. In TLE-TD, the seizures are generally generated in the cerebral cortex surrounding the tumor or in the cortical dysplasia and hence propagate to the hippocampus [18], [77], [78]. The main area of input entry in the hippocampus is the molecular layer of the *fascia dentata* [10], where increased MT-I/II expression was observed in the TLE-TD patients of our study.

Some limitations of our study must be pointed out. So far, studies about MT-I/II expression in animal models of TLE only evaluated the acute period following SE. Considering that our study was performed in patients with chronic epilepsy, it is difficult to establish comparisons between human and animal data. Besides, the reduced number of patients in the TLE-TD group can be the reason why only in one hippocampal subfields the neuronal density correlated with MT-I/II expression. The lack of correlation between seizure frequency and MT-I/II expression does not exclude an association between seizures and MT-I/II expression. Other seizure characteristics, such as seizure duration and time between the last seizure and the surgery, could better correlate with MT-I/II expression than isolated seizure frequency.

Finally, our study may have translational implications in the future. The role of MTs in antiinflamatory response, neurotrophic factor expression, and protection against ROS and heavy metals make those proteins interesting for clinical applications. Studies have shown that EmtinB, a syntethic peptide that mimics the actions of MTs, attenuates kainic acid-induced seizures and protects neurons from excitotoxic death [34]. Further studies with EmtinB and MTs in acute and chronic models of epilepsy might assess, in more detail, the role of these proteins in neuronal survival and seizure susceptibility.

In summary, our data indicate that increased MT-I/II expression is a plastic alteration of chronic TLE, primarily related to the astrogliosis, a common finding in chronic TLE. In opposition to other studies, MT-I/II expression was not associated with significant neuronal survival in TLE. Nevertheless, our findings suggest that increased MT-I/II expression may contribute to the control of the brain hyperexcitability.

## Supporting Information

Figure S1 **Representative images of immunohistochemistries in the temporal cortex from a MTLE patient.** After surgery, tissue fragments were maintained in saline solution during 1 hour (A, F, K and P), 4 hours (B, G, L and Q) and 8 hour (C, H, M and R) prior to fixation in formaline. Note that no difference can be seen regardless of waiting time prior to fixation for MT-I/II (A–C), GFAP (F–H), HLA-DR (K–M) and NeuN (P–R) immunoreactivities. Statistical analyses did not revealed difference in immunopositive area (D, I, N and S) or gray level (E, J, O and T) between tissues fixed after 1 (white boxplot), 2 (very light gray boxplot), 4 (light gray boxplot), 6 (medium gray boxplot) or 8 (dark gray boxplot) hours post surgery for MT-I/II (D and E), GFAP (I and J), HLA-DR (N and O) or NeuN (S and T). Bar in R indicates 100 micrometers.
(TIF)
