# Correction: Increased Metallothionein I/II Expression in Patients with Temporal Lobe Epilepsy

**DOI:** 10.1371/journal.pone.0159122

**Published:** 2016-07-07

**Authors:** José Eduardo Peixoto-Santos, Orfa Yineth Galvis-Alonso, Tonicarlo Rodrigues Velasco, Ludmyla Kandratavicius, João Alberto Assirati, Carlos Gilberto Carlotti, Renata Caldo Scandiuzzi, Luciano Neder Serafini, João Pereira Leite

Fig 2 appears incorrectly in the published article. Please see the correct Fig 2 and its caption below.

**Fig 2:**
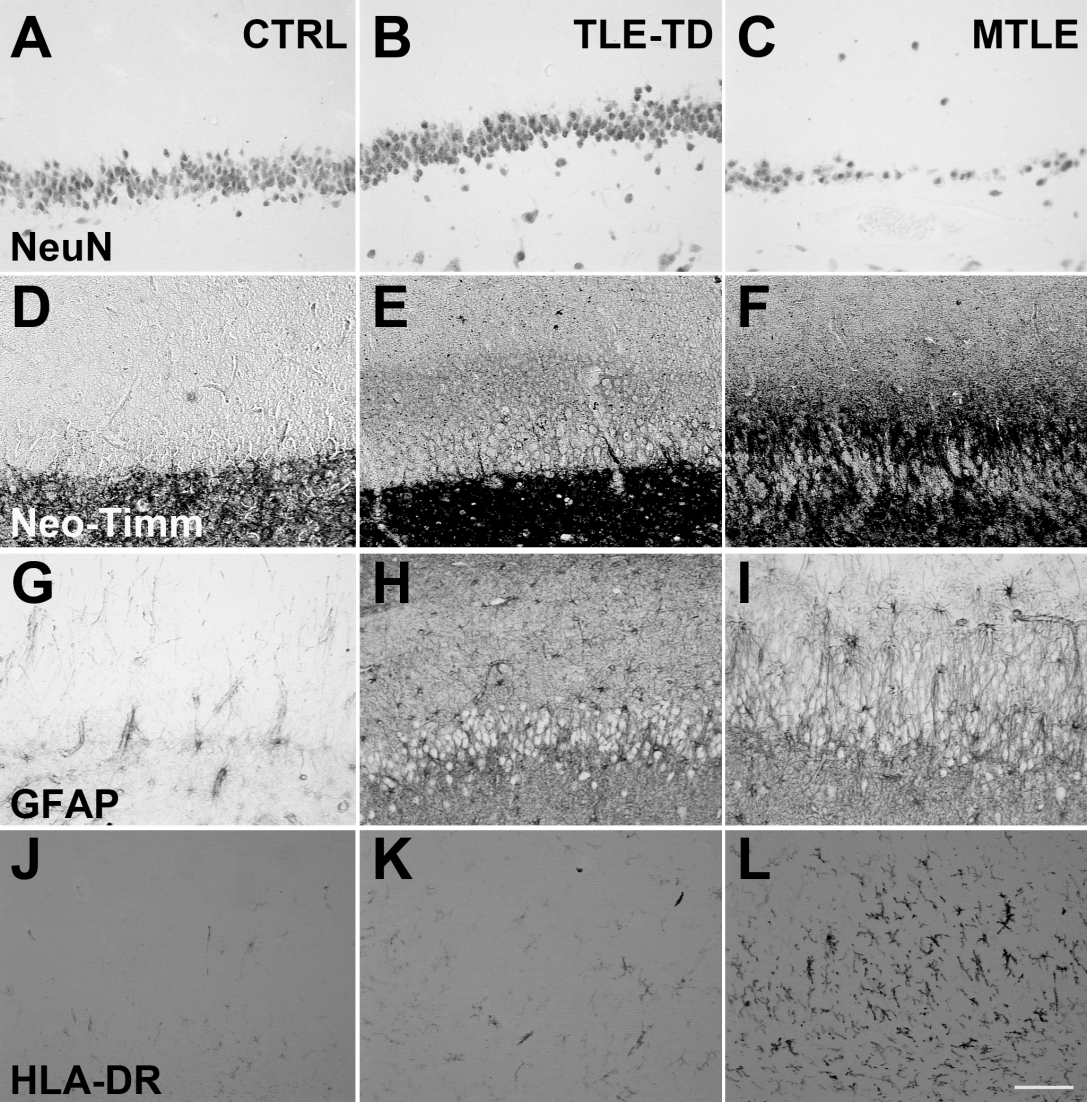
Representative images of NeuN, neo-Timm, GFAP and HLA-DR staining in the Fascia dentata of Ctrl, TLE-TD and MTLE patients. The pattern of NeuN staining is the same in Ctrl (A), TLE-TD (B) and MTLE (C) groups, but MTLE shows reduced neuronal population in this subfield. Compared to Ctrl (D), increased neo-Timm staining was observed in the inner molecular layer of fascia dentata in MTLE patients (F), but not in TLE-TD (E). As for the astroglial population, both hyperplasia and hypertrophy are observed in MTLE (I) and TLE-TD (H), compared to Ctrl (G). Hyperplasia is also observed in microglial cells in TLE-TD (K) and, more notable, in MTLE (L), compared to Ctrl (J). Bar in L indicates 100 micrometers.
