# Supplementary figures and images for: Increased Metallothionein I/II Expression in Patients with Temporal Lobe Epilepsy
Source: PLoS One. 2012 Sep 18;7(9):e44709. doi: 10.1371/journal.pone.0044709 (PMC3445538; doi:10.1371/journal.pone.0044709)

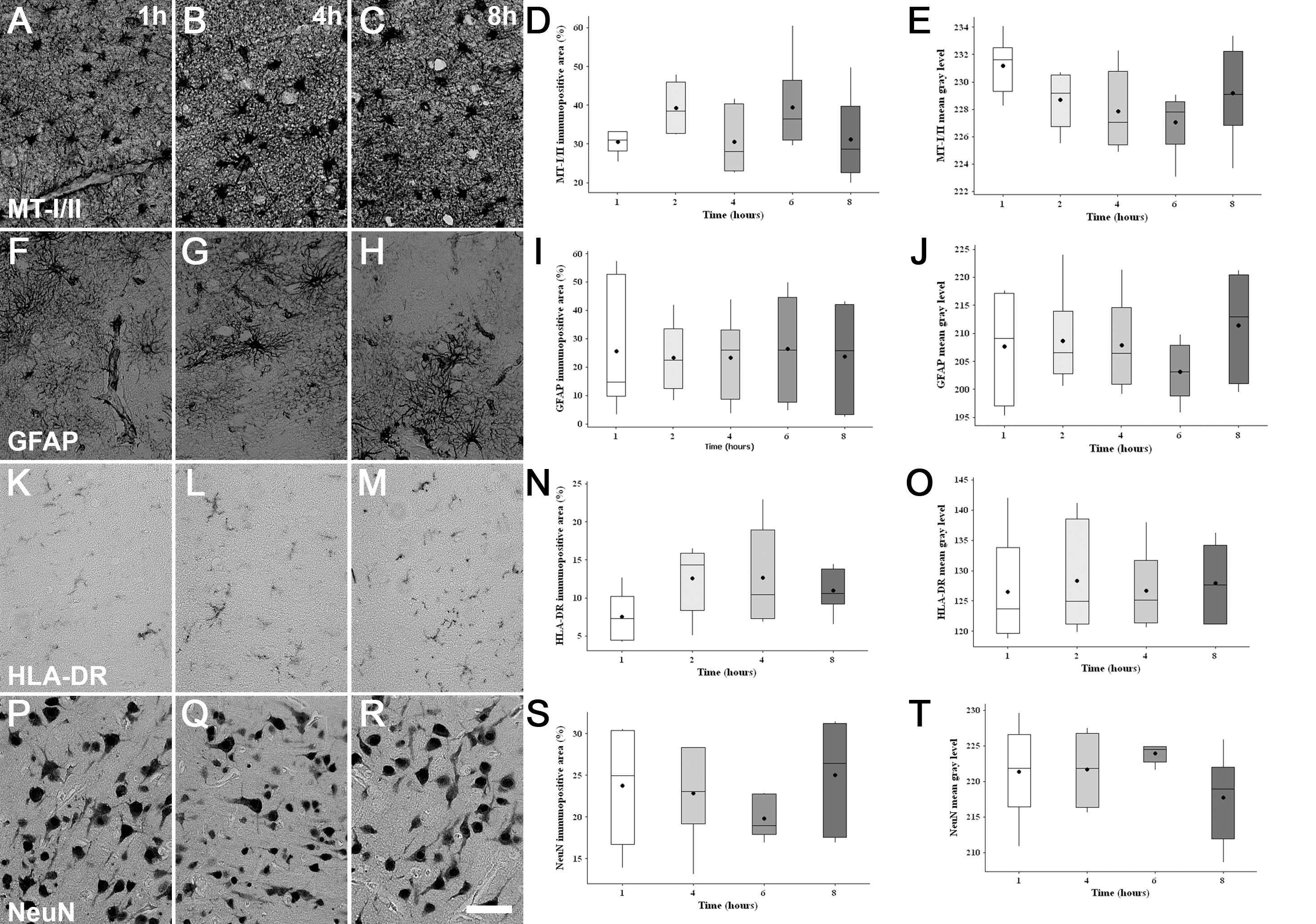

Supplement: Figure S1 — Representative images of immunohistochemistries in the temporal cortex from a MTLE patient. After surgery, tissue fragments were maintained in saline solution during 1 hour (A, F, K and P), 4 hours (B, G, L and Q) and 8 hour (C, H, M and R) prior to fixation in formaline. Note that no difference can be seen regardless of waiting time prior to fixation for MT-I/II (A–C), GFAP (F–H), HLA-DR (K–M) and NeuN (P–R) immunoreactivities. Statistical analyses did not revealed difference in immunopositive area (D, I, N and S) or gray level (E, J, O and T) between tissues fixed after 1 (white boxplot), 2 (very light gray boxplot), 4 (light gray boxplot), 6 (medium gray boxplot) or 8 (dark gray boxplot) hours post surgery for MT-I/II (D and E), GFAP (I and J), HLA-DR (N and O) or NeuN (S and T). Bar in R indicates 100 micrometers. (TIF) [file pone.0044709.s001.tif]
